# Extended middle fossa approach for resection of a petroclival meningioma and vestibular schwannoma

**DOI:** 10.3171/2022.1.FOCVID21258

**Published:** 2022-04-01

**Authors:** Robert C. Rennert, Karol P. Budohoski, Richard K. Gurgel, William T. Couldwell

**Affiliations:** 1 Department of Neurosurgery, Clinical Neurosciences Center, University of Utah, Salt Lake City, Utah; and; 2 Division of Otolaryngology–Head and Neck Surgery, University of Utah, Salt Lake City, Utah

**Keywords:** extended middle fossa, vestibular schwannoma, petroclival meningioma, resection

## Abstract

A 69-year-old woman with refractory left facial pain and subtle left hearing decline had a 13.0 × 8.1–mm left petrous apex/Meckel’s cave meningioma and an 8.8 × 5.6–mm left intracanalicular vestibular schwannoma. She was otherwise neurologically intact. The anterior petrous and middle fossa approaches provide ideal access to these lesions individually, so an extended middle fossa approach was used to resect both in the same setting. She was neurologically stable postoperatively, except for a transient abducens palsy. Hearing was preserved on audiogram, and 4-month MRI displayed no tumors. The extended middle fossa approach provides excellent exposure of the petrous apex and internal auditory canal.

The video can be found here: https://stream.cadmore.media/r10.3171/2022.1.FOCVID21258

## Transcript

### 0:24 Patient History and Imaging.

This is an interesting case of a 69-year-old woman who presented with refractory left facial pain and subtle left hearing loss. She had two lesions, one a petrous apex meningioma extending into Meckel’s cave and the second an intracanalicular Koos grade I vestibular schwannoma.

### 0:45 Surgical Plan and Positioning.

Given the fact that both of these lesions were symptomatic, we’ll perform a single approach for removal of both tumors. We’ll perform a hearing preservation operation through a middle fossa approach and an anterior petrosectomy to remove the meningioma.^1–5^ She’s positioned in the lateral position, and her head is placed in a Mayfield head holder. The reverse-question-mark incision is placed.

### 1:10 Middle Fossa Craniotomy, Drilling of the Internal Auditory Canal, and Anterior Petrosectomy.

We’ll lift off a 4 × 4–cm bone flap centered on the root of the zygoma and identify the foramen spinosum and ligate the middle meningeal artery. We’ll continue our extradural dissection and identify the arcuate eminence and the greater superficial petrosal nerve (GSPN). We’ll proceed with drilling of the internal auditory canal through a middle fossa approach. The canal is identified here, and the dura is preserved. We’ll perform a 270° drilling of the internal auditory canal to enable resection of the vestibular schwannoma. The anterior petrosectomy is then performed. We’ll continue to drill the anterior petrous bone and remove the petrous apex. The petrous apex is then resected.

### 2:33 Removal of Petrous Apex Meningioma.

We’ll then open up the dura over Meckel’s cave and identify the root of the fifth nerve. We then open up over the petrous apex and identify the meningioma extending into Meckel’s cave. We ensure to remove all the dura of the attachment of the meningioma and then dissect the meningioma through the opening. The root of the fifth nerve is visualized.

### 3:11 Removal of Vestibular Schwannoma.

After the meningioma is removed, we’ll turn our attention to the internal auditory canal and resect the vestibular schwannoma. We use auditory brainstem responses for monitoring. The facial nerve is seen here overlying the region of the tumor, and we will ensure that we remove enough of the bone to be able to dissect the tumor completely. We slowly dissect the tumor from the facial nerve and debulk the tumor. The schwannoma is seen here. We define the plane between the nerve and the schwannoma and then, using our microdissectors, elevate the tumor from the region of the cochlear nerve behind it. After resection of the vestibular schwannoma, we test the function of the facial nerve, and the ABR (auditory brainstem response) is preserved.

### 4:14 Closure.

We wax off air cells and place fat with fibrin and glue for closure. The bone flap is replaced with a Medpor cranioplasty. The scalp is closed.

### 4:32 Postoperative Imaging and Clinical Course.

The postoperative imaging demonstrates resection of both lesions. Her postoperative exam was remarkable for a transient left abducens palsy, which resolved over 6 weeks, and her hearing was preserved.

## Acknowledgments

We thank Vance Mortimer for assistance in preparing the surgical video and Kristin Kraus for editorial assistance.

## Disclosures

Dr. Gurgel reports grants from NIH/NIA, Advanced Bionics, and Cochlear LLC; and personal fees from Med-El, outside the submitted work. No other disclosures were reported.

## Author Contributions

Primary surgeon: Couldwell. Assistant surgeon: Gurgel. Editing and drafting the video and abstract: Couldwell, Rennert, Budohoski. Critically revising the work: Rennert, Budohoski, Gurgel. Reviewed submitted version of the work: all authors. Approved the final version of the work on behalf of all authors: Couldwell. Supervision: Couldwell.
